# Evaluation of Influenza Intervention Strategies in Turkey with Fuzzy AHP-VIKOR

**DOI:** 10.1155/2019/9486070

**Published:** 2019-11-19

**Authors:** Funda Samanlioglu

**Affiliations:** Department of Industrial Engineering, Kadir Has University, 34083 Cibali, Istanbul, Turkey

## Abstract

In this study, a fuzzy AHP-VIKOR method is presented to help decision makers (DMs), especially physicians, evaluate and rank intervention strategies for influenza. Selecting the best intervention strategy is a sophisticated multiple criteria decision-making (MCDM) problem with potentially competing criteria. Two fuzzy MCDM methods, fuzzy analytic hierarchy process (F-AHP) and fuzzy VIsekriterijumska optimizacija i KOmpromisno Resenje (F-VIKOR), are integrated to evaluate and rank influenza intervention strategies. In fuzzy AHP-VIKOR, F-AHP is used to determine the fuzzy criteria weights and F-VIKOR is implemented to rank the strategies with respect to the presented criteria. A case study is given where a professor of infectious diseases and clinical microbiology, an internal medicine physician, an ENT physician, a family physician, and a cardiologist in Turkey act as DMs in the process.

## 1. Introduction

The 2009 A(H1N1) influenza pandemic caused a global alert, and all countries implemented various intervention strategies. Some measures across communities were pharmaceutical such as antivirals and vaccination and some were nonpharmaceutical such as limiting public gatherings, closing schools, and restricting travel [1, 2]. Union Health Security Committee recommended to vaccinate risk and target groups such as pregnant women, healthcare workers, and people older than six months with chronic illnesses [3, 4]. Unless an effective intervention strategy is applied, influenza spreads rapidly in seasonal epidemics and costs society a substantial amount in terms of healthcare expenses, lost productivity, and loss of life.

During the 2009 A(H1N1) influenza pandemic, in EU, Hungary started vaccination first, and by July 2010, about 9% was vaccinated in EU/EEA [3]. However, in most of the countries, vaccination campaigns were not as effective as planned due to the timing and the percentage of coverage [5]. Norway and Sweden were compared in terms of their vaccination strategies in a previous study [5]. In Sweden, vaccination campaign was more effective than Norway. Even though vaccination started almost the same time in both countries and although about 40% of population got vaccinated, in Norway, it was too late to be effective due to the relative timing of the starting time of vaccination and its location in the epidemic wave [5–7]. As discussed in Samanlioglu and Bilge's study [5], for the vaccination campaign to be effective, vaccination should start in the early phases of the epidemic, but it does not need to continue over the peak of the epidemic. The effect of vaccination timing and sales of antivirals in Norway were analysed, and they showed that the countermeasures only prevented 11-12% of the potential cases relative to an unmitigated pandemic, and that if the campaign would have started 6 weeks earlier, the vaccination alone might have reduced the clinical attack rate by 50% [6]. The interventions in France and Germany were discussed in a previous study, and even though Germany and France have similar vaccination policies, the relative fatalities were higher in France [5]. The peak of the epidemic was delayed in France due to the timing of school holidays [8]. The difference can be explained by epidemic-specific precautions and healthcare procedures applied in Germany [9].

As realized from 2009 A(H1N1) pandemic, a systematic approach is needed for effective health planning and making decisions related to intervention strategies during an influenza pandemic, especially for transparency and accountability of the decision-making process. Evaluation of intervention strategies is a significant MCDM problem that requires expertise and competency since there are various potentially conflicting criteria to take into consideration. In the literature, there are a few studies that utilize MCDM methods for evaluation of intervention strategies. Shin et al. [10] used AHP to evaluate the expanded Korean immunization programs and assess two policies: weather private clinics and hospitals or public health centers should offer free vaccination services to children. Mourits et al. [11] applied the EVAMIX (evaluations with mixed data) MCDM method to rank alternative strategies to control classical swine fever epidemics in EU. Aenishaenslin et al. [12] implemented D-Sight which uses PROMETHEE methods (Preference Ranking Organization Method for Enrichment Evaluations) and gives access to the GAIA (Geometrical Analysis for Interactive Aid) to assess various prevention and control strategies for the Lyme disease in Quebec, Canada. They developed two MCDM models, one for surveillance interventions and one for control interventions, and conducted the analysis under a disease emergence and an epidemic scenario. Pooripussarakul et al. [13] implemented best-worst scaling to assess and rank-order vaccines for introduction into the expanded program on immunization in Thailand.

In this study, various influenza intervention strategies are evaluated, taking into consideration potentially conflicting criteria, by five physicians with different expertises acting as consultants and decision makers (DMs). As the MCDM method and integrated method, fuzzy AHP-VIKOR is implemented to evaluate and rank the strategies. In fuzzy AHP-VIKOR, F-AHP is implemented to find the fuzzy criteria weights and F-VIKOR is utilized to rank alternatives using these weights. Here, an integrated method is used to have both methods' advantages. F-VIKOR is easy to use for MCDM problems with especially conflicting criteria; however, it does not include guidelines for determining the weights of criteria, and with F-AHP, through pairwise comparisons, reliable fuzzy weights can be obtained. With the integrated fuzzy AHP-VIKOR, intervention strategies are ranked without too many repetitive pairwise comparisons and complicated calculations.

The fuzzy set theory is a mathematical theory designed to model the vagueness or imprecision of human cognitive processes. It is a theory of classes with unsharp boundaries, and any crisp theory can be fuzzified by generalizing the concept of a set within that theory to the concept of a fuzzy set [14]. Fuzzy extensions of AHP (F-AHP) and VIKOR (F-VIKOR) are used to capture the uncertainty and vagueness on judgments of DMs.

In AHP [15], alternatives are evaluated based on various criteria in a hierarchical and multilevel structure, and then alternatives are ranked based on a calculated total weighted score. AHP is used widely in real-life applications, i.e., for decisions related to machine shops [16], for evaluation of machine tools [17, 18], and for evaluation of medical devices and materials [19]. The VIKOR method was introduced mainly for MCDM problems with competing or noncommensurable criteria. In VIKOR, compromise ranking is performed, and alternatives are compared according to the closeness to the ideal solution [20–23]. To reflect the uncertainty and vagueness on judgments of DMs, their fuzzy extensions, F-AHP and F-VIKOR, have been developed. With F-VIKOR, an accepted compromise solution is obtained with a maximum group utility of the majority and a minimum of individual regret of the opponent [22, 24]. In the literature, different versions of F-VIKOR exist such as F-VIKOR with: Triangular fuzzy numbers [24, 25], triangular intuitionistic fuzzy numbers [26], 2-tuple group decision-making linguistic model [27], an attitudinal-based interval 2-tuple linguistic model [28], type-2 fuzzy model [29, 30], and an intuitionistic hesitant model using entropy weights [31]. Several real-life applications of F-AHP, F-VIKOR, and fuzzy AHP-VIKOR are given in Table 1.

At present, there does not appear to be a research paper in the literature that focuses on evaluation and ranking of influenza intervention strategies. Moreover, fuzzy AHP-VIKOR has never been used in the evaluation of intervention strategies for a pandemic. In the next sections, fuzzy AHP-VIKOR steps and a case study are presented.

## 2. Proposed Fuzzy AHP-VIKOR Approach

### 2.1. Definitions

In fuzzy set theory, there are classes with unsharp boundaries [61, 62]. Any crisp theory can be fuzzified using the concept of a fuzzy set [14]. In the proposed fuzzy AHP-VIKOR, triangular fuzzy numbers (TFNs) are used due to its simplicity. A fuzzy number is a special fuzzy set *F*={(*x*, *μ*_*F*_(*x*)), *x* ∈ *R*}. Here, *R* : −*∞* < *x* < +*∞* and *μ*_*F*_(*x*) is from *R* to [0, 1]. A TFN denoted as $\tilde{M}=\left(l,m,u\right)$, where *l* ≤ *m* ≤ *u*, has the membership function:

(1) $$\begin{array}{c} \mu_{F}\left(x\right)=\left\{\begin{array}{cc} 0, & x<l,\\ \frac{x-l}{m-l}, & l\leq x\leq m,\\ \frac{u-x}{u-m}, & m\leq x\leq u,\\ 0, & x>u. \end{array}\right. \end{array}$$

Basic operations between two positive TFNs $\tilde{A}=\left(l_{1},m_{1},u_{1}\right)$, $\tilde{B}=\left(l_{2},m_{2},u_{2}\right)$, *l*_1_ ≤ *m*_1_ ≤ *u*_1_, *l*_2_ ≤ *m*_2_ ≤ *u*_2_, and a crisp number *C*(*C* ≥ 0) are explained as follows:

(2) $$\begin{array}{c} \tilde{A}+\tilde{B}=\left(l_{1}+l_{2},m_{1}+m_{2},u_{1}+u_{2}\right),\\ \tilde{A}+C=\left(l_{1}+C,m_{1}+C,u_{1}+C\right),\\ \tilde{A}-\tilde{B}=\left(l_{1}-u_{2},m_{1}-m_{2},u_{1}-l_{2}\right),\\ \tilde{A}-C=\left(l_{1}-C,m_{1}-C,u_{1}-C\right),\\ \tilde{A}*\tilde{B}=\left(l_{1}*l_{2},m_{1}*m_{2},u_{1}*u_{2}\right),\\ \tilde{A}*C=\left(l_{1}*C,m_{1}*C,u_{1}*C\right),\\ \frac{\tilde{A}}{\tilde{B}}=\left(\frac{l_{1}}{u_{2}},\frac{m_{1}}{m_{2}},\frac{u_{1}}{l_{2}}\right),\\ \frac{\tilde{A}}{\tilde{B}}=\left(\min\left(\frac{l_{1}}{l_{2}},\frac{l_{1}}{u_{2}},\frac{u_{1}}{l_{2}},\frac{u_{1}}{u_{2}}\right),\frac{m_{1}}{m_{2}},\max\left(\frac{l_{1}}{l_{2}},\frac{l_{1}}{u_{2}},\frac{u_{1}}{l_{2}},\frac{u_{1}}{u_{2}}\right)\right)\text{,}\;\text{if }\tilde{A}\text{ and }\tilde{B}\text{ are TFNs }\left(\text{not neccesarily positive TFNs}\right),\\ \frac{\tilde{A}}{C}=\left(\frac{l_{1}}{C},\frac{m_{1}}{C},\frac{u_{1}}{C}\right)\text{,}\;\text{for }C>0,\\ {\tilde{A}}^{-1}=\left(\frac{1}{u_{1}},\frac{1}{m_{1}},\frac{1}{l_{1}}\right),\\ \text{max}\left(\tilde{A}+\tilde{B}\right)=\left(\max\left(l_{1},l_{2}\right),\max\left(m_{1},m_{2}\right),\max\left(u_{1},u_{2}\right)\right),\\ \text{min}\left(\tilde{A}+\tilde{B}\right)=\left(\min\left(l_{1},l_{2}\right),\min\left(m_{1},m_{2}\right),\min\left(u_{1},u_{2}\right)\right). \end{array}$$

The graded mean integration approach [63] is used as the defuzzification method to convert TFNs into crisp numbers. Here,

(3) $$\begin{array}{c} \text{crisp}\left(\tilde{A}\right)=\frac{\left(4m_{1}+l_{1}+u_{1}\right)}{6}. \end{array}$$

### 2.2. Finding the Important Weights of Criteria with F-AHP

After constructing the hierarchical structure of the problem, the DMs make pairwise comparisons of the criteria and estimate their relative importance in relation to the element at the immediate proceeding level. During the process of evaluation of criteria, the pairwise comparisons are made by using the linguistic terms and scale presented in Table 2.

#### 2.2.1. Computational Steps of F-AHP

**Step 1 .**

Form a decision group of *K* people. Identify *n* criteria and select the suitable linguistic terms for the pairwise comparison of criteria. Calculate the aggregated ${\tilde{x}}_{ij}=\left(1/K\right)\left({\tilde{x}}_{ij}^{1}\left(+\right){\tilde{x}}_{ij}^{2}\left(+\right)\cdots \left(+\right){\tilde{x}}_{ij}^{K}\right)$ where ${\tilde{x}}_{ij}^{K}=\left(a_{ij}^{K},b_{ij}^{K},c_{ij}^{K}\right),\text{ in which }\forall i,j,\;k$ is the TFN corresponding to the evaluation of the *K*^th^ DM.

**Step 2 .**

$\tilde{X}=\left[\begin{array}{ccccc} \left(1,1,1\right) & {\tilde{x}}_{12} & \cdots & \cdots & {\tilde{x}}_{1n}\\ {\tilde{x}}_{21} & \left(1,1,1\right) & \cdots & \cdots & {\tilde{x}}_{2n}\\ \cdots & \cdots & \cdots & \cdots & \cdots \\ \cdots & \cdots & \cdots & \cdots & \cdots \\ {\tilde{x}}_{n1} & {\tilde{x}}_{n2} & \cdots & \cdots & \left(1,1,1\right) \end{array}\right]$ with elements ${\tilde{x}}_{ij}=\left(a_{ij},b_{ij},c_{ij}\right)$ is normalized and $\tilde{S}$ is obtained. $\tilde{S}=\left[\begin{array}{ccccc} {\tilde{s}}_{11} & {\tilde{s}}_{12} & \cdots & \cdots & \tilde{s_{1n}}\\ {\tilde{s}}_{21} & {\tilde{s}}_{22} & \cdots & \cdots & {\tilde{s}}_{2n}\\ \cdots & \cdots & \cdots & \cdots & \cdots \\ \cdots & \cdots & \cdots & \cdots & \cdots \\ {\tilde{s}}_{n1} & {\tilde{s}}_{n2} & \cdots & \cdots & {\tilde{s}}_{nn} \end{array}\right]$, where ${\tilde{s}}_{ij}=\left(a_{ij}/\sum_{i}c_{ij},b_{ij}/\sum_{i}b_{ij},c_{ij}/\sum_{i}a_{ij}\right)$. Fuzzy priority weight vector ${\tilde{w}}_{\text{criteria}}=\left({\tilde{w}}_{1},{\tilde{w}}_{2},\ldots ,{\tilde{w}}_{n}\right)$ is calculated by averaging the entries on each row of $\tilde{S}$.

**Step 3 .**

$\tilde{X}$ is defuzzified by using equation (3), and *w*_cr_=(*w*_1_, *w*_2_,…, *w*_*n*_) (approximate crisp criteria weights) is calculated by averaging the entries on each row of normalized *X*. So the normalized principal eigen vector is *w*_cr_. The largest eigenvalue, called the principal eigenvalue (*λ*_max_), is determined with the following equation:

(4) $$\begin{array}{c} Xw_{\text{cr}}^{T}=\lambda_{\max}w_{\text{cr}}^{T}. \end{array}$$

The measure of inconsistency of pairwise comparisons is called the consistency index (CI), and it is calculated as

(5) $$\begin{array}{c} \text{CI}=\frac{\lambda_{\max}-n}{n-1}. \end{array}$$

The consistency ratio (CR) is used to estimate the consistency of pairwise comparisons, and the CR is calculated by dividing CI by the random consistency index (RI):

(6) $$\begin{array}{c} \text{CR}=\frac{\text{CI}}{\text{RI}}. \end{array}$$

RI is the average index for randomly generated weights [15]. If the CR is less than 0.10, the comparisons are acceptable; otherwise, they are not.

### 2.3. Ranking of Alternatives with F-VIKOR

In the previous section, fuzzy priority weight vector ${\tilde{w}}_{\text{criteria}}=\left({\tilde{w}}_{1},{\tilde{w}}_{2},\ldots ,{\tilde{w}}_{n}\right)$ was obtained with F-AHP. After the determination of ${\tilde{w}}_{\text{criteria}}$ with F-AHP, in order to rank the alternatives, F-VIKOR is used. During the process of evaluation of alternatives with F-VIKOR, the linguistic terms and scale presented in Table 3 is used.

#### 2.3.1. Computational Steps of F-VIKOR

**Step 4 .**

Identify the *m* alternatives and select the suitable linguistic terms for the evaluations of alternatives with respect to each criterion. Calculate the aggregated ${\tilde{r}}_{ij}=\left(1/K\right)\left({\tilde{r}}_{ij}^{1}\left(+\right){\tilde{r}}_{ij}^{2}\left(+\right)\cdots \left(+\right){\tilde{r}}_{ij}^{K}\right)$ where ${\tilde{r}}_{ij}^{K}=\left(a_{ij}^{K},b_{ij}^{K},c_{ij}^{k}\right)$ is the TFN for the evaluation of the *K*^th^ DM. After the aggregation, the fuzzy MCDM problem with *m* alternatives that are evaluated in terms of *n* criteria can be expressed in a fuzzy matrix format as $\tilde{D}=\left[\begin{array}{ccccc} {\tilde{r}}_{11} & {\tilde{r}}_{12} & \cdots & \cdots & {\tilde{r}}_{1n}\\ {\tilde{r}}_{21} & {\tilde{r}}_{22} & \cdots & \cdots & {\tilde{r}}_{2n}\\ \cdots & \cdots & \cdots & \cdots & \cdots \\ \cdots & \cdots & .. & \cdots & \cdots \\ {\tilde{r}}_{n1} & {\tilde{r}}_{n2} & \cdots & \cdots & {\tilde{r}}_{mn} \end{array}\right]$, where ${\tilde{r}}_{ij}=\left(a_{ij},b_{ij},c_{ij}\right),\;\forall i,j$, are positive TFNs.

**Step 5 .**

Find the fuzzy best value (FBV; ${\tilde{f}}_{j}^{*}$) and the fuzzy worst value (FWV; ${\tilde{f}}_{j}^{-}$) for each criterion:

(7) $$\begin{array}{c} {\tilde{f}}_{j}^{*}=\underset{i}{\max}{\tilde{r}}_{ij},\;\forall j,\\ {\tilde{f}}_{j}^{-}=\underset{i}{\min}{\tilde{r}}_{ij},\;\forall j. \end{array}$$

**Step 6 .**

Calculate the separation measures of each alternative from the FBV (${\tilde{S}}_{i}$) and FWV (${\tilde{R}}_{i}$):

(8) $$\begin{array}{c} {\tilde{S}}_{i}=\overset{n}{\underset{j=1}{\sum }}\frac{{\tilde{w}}_{j}\left({\tilde{f}}_{j}^{*}-{\tilde{r}}_{ij}\right)}{\left({\tilde{f}}_{j}^{*}-{\tilde{f}}_{j}^{-}\right)},\;\forall i,\\ {\tilde{R}}_{i}=\underset{j}{\max}\left[\frac{{\tilde{w}}_{j}\left({\tilde{f}}_{j}^{*}-{\tilde{r}}_{ij}\right)}{{\tilde{f}}_{j}^{*}-{\tilde{f}}_{j}^{-}}\right],\;\forall i. \end{array}$$

**Step 7 .**

Calculate ${\tilde{S}}^{*},{\tilde{S}}^{-},{\tilde{R}}^{*},\text{ and }{\tilde{R}}^{-}$ values as

(9) $$\begin{array}{c} {\tilde{S}}^{*}=\underset{i}{\min}{\tilde{S}}_{i},\\ {\tilde{S}}^{-}=\underset{i}{\max}{\tilde{S}}_{i},\\ {\tilde{R}}^{*}=\underset{i}{\min}{\tilde{R}}_{i},\\ {\tilde{R}}^{-}=\underset{i}{\max}{\tilde{R}}_{i}. \end{array}$$

**Step 8 .**

Calculate ${\tilde{Q}}_{i}$ values for each alternative:

(10) $$\begin{array}{c} {\tilde{Q}}_{i}=\nu \frac{{\tilde{S}}_{i}-{\tilde{S}}^{*}}{{\tilde{S}}^{-}-{\tilde{S}}^{*}}+\left(1-\nu \right)\frac{{\tilde{R}}_{i}-{\tilde{R}}^{*}}{{\tilde{R}}^{-}-{\tilde{R}}^{*}},\;\forall i, \end{array}$$

where *ν* is the weight of the strategy of the maximum group utility (majority of criteria) and 1 − *ν* is the weight of the individual regret. *ν* is usually assumed to be 0.5 (by consensus) [52, 57].

**Step 9 .**

Defuzzify the ${\tilde{Q}}_{i}$ values with equation (3) and rank the alternatives based on crisp *Q*_*i*_ values. Consequently, the smaller the *Q*_*i*_, the better the alternative.

**Step 10 .**

Determine a compromise solution. Assume that two conditions below are acceptable. Then, by using *Qi*, a single optimal solution *A*^(1)^ is determined.

**Condition 1 (acceptable advantage).**

*Q*(*A*^(2)^) − *Q*(*A*^(1)^) ≥ *DQ* and *DQ* = 1/(*m* − 1) but *DQ* = 0.25 if *m* < 4. Here, *A*^(1)^ is the first ranked alternative and *A*^(2)^ is the second ranked alternative based on crisp *Q*_*i*_ values, and *m* is the number of alternatives.

**Condition 2 (acceptable stability in decision-making).**

*Q*(*A*^(1)^) must be *S*(*A*^(1)^) and/or *R*(*A*^(1)^) under this condition.

If Condition 1 is not accepted and *Q*(*A*^(*m*)^) − *Q*(*A*^(1)^) < *DQ*, then *A*^(*m*)^ and *A*^(1)^ are the same compromise solution. *A*^(1)^ does not have a comparative advantage, so the compromise solutions *A*^(1)^, *A*^(2)^,…, *A*^(*m*)^ are the same. If Condition 2 is not accepted, the stability of decision-making is deficient although *A*^(1)^ has a comparative advantage. Hence, compromise solutions *A*^(1)^ and *A*^(2)^ are same [51, 64, 65].

## 3. Case Study

In this study, DMs are a professor of infectious diseases and clinical microbiology (DM1), an internal medicine physician (DM2), an ENT physician (DM3), a family physician (DM4), and a cardiologist (DM5) in Turkey. 8 benefit criteria are determined by the DMs for the evaluation of influenza intervention strategies. These are listed in Table 4.

The alternatives that are going to be ranked are mass vaccination (A1), antiviral treatment and isolation of infected individuals (A2), and exclusion of people from high risk areas (mass measurements to reduce the contact rate, i.e. school closures, and closure of public places) (A3).

In order to determine the fuzzy criteria weights, F-AHP is used. In F-AHP, first DMs do pairwise comparison of criteria using the linguistic terms presented in Table 2. Comparisons of 5 DMs are presented in Table 5. After the aggregation of the corresponding TFNs of the DMs evaluations, in Table 6, $\tilde{X}$ is given. Afterwards, fuzzy priority weight vector ${\tilde{w}}_{\text{criteria}}=\left({\tilde{w}}_{1},{\tilde{w}}_{2},\ldots ,{\tilde{w}}_{n}\right)$ is calculated by averaging the entries on each row of normalized $\tilde{X}\left(\tilde{S}\right)$. ${\tilde{w}}_{\text{criteria}}$ is presented in Table 7. In order to calculate the CR of $\tilde{X}$, equation (3) is utilized for defuzzification. CR is determined as 0.0483, and since it is less than 0.1, the comparison results are considered to be consistent.

${\tilde{w}}_{\text{criteria}}=\left({\tilde{w}}_{1},{\tilde{w}}_{2},\ldots ,{\tilde{w}}_{n}\right)$ determined with F-AHP is used in F-VIKOR to rank intervention alternatives. In F-VIKOR, first DMs evaluate alternatives with respect to evaluation criteria using the linguistic terms presented in Table 3. These evaluations are presented in Table 8. After the aggregation of the corresponding TFNs of the DMs' evaluations, in Table 9, $\tilde{D}$ is presented. Also, in Table 9, the FBV (${\tilde{f}}_{j}^{*}$) and the FWV (${\tilde{f}}_{j}^{-}$) for each criterion are presented. The separation measures of each alternative ${\tilde{S}}_{i}$ and ${\tilde{R}}_{i}$ are given in Table 10, along with ${\tilde{S}}^{*},{\tilde{S}}^{-},{\tilde{R}}^{*},\text{ and }{\tilde{R}}^{-}$ values. Based on these, ${\tilde{Q}}_{i}$ value for each alternative is calculated and presented in Table 10. Afterwards, ${\tilde{Q}}_{i}$, ${\tilde{S}}_{i}$, and ${\tilde{R}}_{i}$ values are defuzzified with equation (3), and ranking of alternatives with respect to *S*_*i*_, *R*_*i*_,  and *Q*_*i*_ are shown in Table 11.

Consequently, the smaller the *Q*_*i*_, the better the alternative, so based on *Q*_*i*_, alternatives are ranked from best to worst as mass vaccination (A1), antiviral treatment and isolation of infected individuals (A2), and exclusion of people from high risk areas (mass measurements to reduce the contact rate, i.e., school closures, closure of public places, etc.) (A3). However, to determine a compromise solution, Conditions 1 and 2 are checked. Condition 1 (acceptable advantage) is not satisfied when A1 and A2 are compared since *Q*(*A*^(2)^) − *Q*(*A*^(1)^)=0.171 − 0.085=0.086 < *DQ*=0.25. Condition 2 (acceptable stability in decision-making) is satisfied since *Q*(*A*^(1)^) is also *R*(*A*^(1)^), as shown in Table 11. Compromise solutions A1 and A2 are the same. Since *Q*(*A*^(3)^) − *Q*(*A*^(1)^)=0.639 − 0.085=0.555 ≥ *DQ*=0.25, A3 and A1 are not the same compromise solution and A1 has acceptable advantage over A3. Also, A1 is better ranked than A3 in terms of *S*_*i*_ and *R*_*i*_ values, as shown in Table 11, so there is acceptable stability in decision-making. Since *Q*(*A*^(3)^) − *Q*(*A*^(2)^)=0.639 − 0.171=0.468 ≥ *DQ*=0.25, A3 and A2 are not the same compromise solution and A2 has acceptable advantage over A3. Also, A2 is better ranked than A3 in terms of *S*_*i*_ and *R*_*i*_ values, as shown in Table 11, so there is acceptable stability in decision-making.

Although based on *Q*_*i*_ values A1 is better ranked than A2, A1 does not have comparative advantage over A2, so compromise solutions A1 and A2 are same and they both have comparative advantage over A3. So, based on these evaluations and calculations, mass vaccination strategy and antiviral treatment and isolation of infected individuals strategy are found to be the best intervention strategies with no reasonable difference, and exclusion of people from high risk areas strategy is determined to be worse than both of these strategies.

## 4. Conclusions

In this study, the results of a multicriteria decision analysis for effective management of a health issue-influenza are presented. More specifically, in this research, an integrated fuzzy AHP-VIKOR method is implemented to evaluate influenza intervention strategies. At present, there does not appear to be a MCDA in the literature for the evaluation of influenza intervention strategies. Expert opinion for the development of pairwise comparison matrices of criteria and evaluation of alternatives was needed in the fuzzy AHP-VIKOR method, so a professor of infectious diseases and clinical microbiology, an internal medicine physician, an ENT physician, a family physician, and a cardiologist in Turkey acted as DMs in the study. Based on their evaluation, mass vaccination and antiviral treatment and isolation of infected individuals are determined as the best intervention strategies with no comparative advantage and exclusion of people from high risk areas (mass measurements to reduce the contact rate, i.e., school closures, and closure of public places) is determined to be the worst alternative among the evaluated.

For future research, the proposed fuzzy AHP-VIKOR method and determined evaluation criteria can be adopted and utilized by physicians for the evaluation and ranking of intervention strategies for similar diseases. Also, outer dependence, innerdependence, and feedback relationships between evaluation criteria can be investigated with the fuzzy analytic network process (F-ANP), and F-ANP can be integrated with F-VIKOR for healthcare-related evaluation and ranking problems such as drug selection and treatment selection.

## Figures and Tables

**Table 1:** Fuzzy AHP, fuzzy VIKOR, and fuzzy AHP-VIKOR applications.

Fuzzy AHP applications	(i) Selection of concepts in an NPD environment [32]
	(ii) Evaluation of machine tools in a manufacturing system [33, 34]
	(iii) Evaluation of notebook computers for buyers [35]
	(iv) Evaluation of disassembly line balancing solutions [36]
	(v) Selection of power substation locations [37]
	(vi) Selection of thermal power plant locations [38]
	(vii) Selection of biodiesel blend for IC engines [39]

Fuzzy VIKOR applications	(i) Water resources planning [25]
	(ii) Evaluation of the vulnerability of the water supply to climate change and variability in South Korea [40]
	(iii) Material selection in an engineering application [41]
	(iv) Reverse logistics [42]
	(v) Site selection in waste management [28]
	(vi) Evaluation of hospital services in Taiwan [43]
	(vii) Selection of CNC machine tools [44]
	(viii) Evaluation of schools' academic performance [45]
	(ix) Selection of green supplier development programs [46]
	(x) Review papers about VIKOR and fuzzy VIKOR applications [47, 48]
	(xi) Selection of a managed security service provider [49]
	(xii) Selection of measures for prevention and reduction of “smog” (smoke and fog) in Pakistan [50]
	(xiii) Risk assessment of China-Pakistan fiber optic project (CPFOP) [51]

Fuzzy AHP-VIKOR applications	(i) Selection of the best renewable energy alternative and the best energy production site for Istanbul [52]
	(ii) Selection of machine tool alternative for the manufacturing sector [53]
	(iii) Evaluation of the performance levels of Turkish banks registered in Borsa Istanbul (AHP and F-VIKOR) [54]
	(iv) Ranking the financial performance of several Iranian companies [55]
	(v) Evaluation of performance of Iranian cement firms (F-AHP and VIKOR) [56]
	(vi) Selection of pipe material in sugar industry (F-AHP and VIKOR) [57]
	(vii) Evaluation of busses for public transportation [58]
	(viii) Selection of the best knowledge flow practicing organization [59]
	(ix) Evaluation of compliant polishing tool (AHP and F-VIKOR) [60]

**Table 2:** Linguistic terms and TFNs for the evaluation of criteria in F-AHP.

Linguistic terms	Triangular fuzzy number (TFN)
Absolutely strong (AS)	(2, 5/2, 3)
Very strong (VS)	(3/2, 2, 5/2)
Fairly strong (FS)	(1, 3/2, 2)
Slightly strong (SS)	(1, 1, 3/2)
Equal (E)	(1, 1, 1)
Slightly weak (SW)	(2/3, 1, 1)
Fairly weak (FW)	(1/2, 2/3, 1)
Very weak (VW)	(2/5, 1/2, 2/3)
Absolutely weak (AW)	(1/3, 2/5, 1/2)

**Table 3:** Linguistic terms and TFNs for the ratings of alternatives in F-VIKOR.

Linguistic terms	Triangular fuzzy number (TFN)
Very poor (VP)	(0, 0, 1)
Poor (P)	(0, 1, 3)
Medium poor (MP)	(1, 3, 5)
Fair (F)	(3, 5, 7)
Medium good (MG)	(5, 7, 9)
Good (G)	(7, 9, 10)
Very good (VG)	(9, 10, 10)

**Table 4:** Evaluation criteria for influenza intervention strategies.

C1	Effectiveness (reduction of incidence of cases)
C2	Lack of health side effects
C3	Cost-effectiveness
C4	Feasibility and timing (minimum delay before results)
C5	Public acceptance
C6	Equity and availability (proportion of population benefitting)
C7	Applicability (easiness and minimum complexity)
C8	Lack of unintended effects about work and social life

**Table 5:** 5 DMs' pairwise comparison of evaluation criteria.

	C1	C2	C3	C4	C5	C6	C7	C8
C1	E	AS	VS	VS	AS	SS	VS	FS
	E	AS	FS	VS	VS	SS	VS	VS
	E	E	FS	VS	SS	E	E	SS
	E	VS	E	SW	VW	E	SW	E
	E	AS	S	VS	VS	E	E	SS

C2		E	FS	VS	FS	FS	FS	FS
		E	FS	FS	SS	SS	SS	E
		E	FS	SS	AS	SW	FW	SW
		E	VS	SS	SS	SS	E	FS
		E	FS	FS	AS	SS	FS	FS

C3			E	FS	VW	FS	E	E
			E	FS	SS	FS	E	FS
			E	E	VS	E	SW	VW
			E	FS	SW	E	E	FS
			E	SS	FW	SS	E	E

C4				E	FW	FS	E	E
				E	SW	VS	SS	E
				E	SS	E	SS	FW
				E	SW	SW	SW	SW
				E	SW	FS	E	E

C5					E	VS	FS	FS
					E	AS	FS	VS
					E	VS	VS	VS
					E	FW	FS	FS
					E	VS	VS	SS

C6						E	E	E
						E	SS	E
						E	E	VW
						E	FS	FS
						E	E	E

C7							E	E
							E	SS
							E	E
							E	VS
							E	E

C8								E
								E
								E
								E
								E

**Table 6:** Fuzzy evaluation matrix for the criteria weights ($\tilde{X}$).

	C1	C2	C3	C4	C5	C6	C7	C8
C1	(1.000, 1.000, 1.000)	(1.700, 2.100, 2.500)	(0.900, 1.200, 1.500)	(1.334, 1.800, 2.200)	(1.280, 1.600, 2.034)	(1.000, 1.000, 1.200)	(1.134, 1.400, 1.600)	(1.100, 1.300, 1.700)
C2	(0.478, 0.540, 0.634)	(1.000, 1.000, 1.000)	(1.100, 1.600, 2.100)	(1.100, 1.400, 1.900)	(1.400, 1.700, 2.200)	(0.934, 1.100, 1.500)	(0.900, 1.134, 1.500)	(0.934, 1.300, 1.600)
C3	(0.480, 0.568, 0.734)	(0.480, 0.636, 0.934)	(1.000, 1.000, 1.000)	(1.000, 1.300, 1.700)	(0.814, 1.034, 1.334)	(1.000, 1.200, 1.500)	(0.934, 1.000, 1.000)	(0.880, 1.100, 1.334)
C4	(0.520, 0.600, 0.836)	(0.548, 0.768, 0.934)	(0.634, 0.802, 1.000)	(1.000, 1.000, 1.000)	(0.702, 0.934, 1.100)	(1.034, 1.400, 1.700)	(0.934, 1.000, 1.200)	(0.834, 0.934, 1.000)
C5	(0.660, 0.880, 1.068)	(0.500, 0.694, 0.800)	(0.914, 1.200, 1.534)	(0.934, 1.100, 1.500)	(1.000, 1.000, 1.000)	(1.400, 1.834, 2.300)	(1.200, 1.700, 2.200)	(1.200, 1.600, 2.100)
C6	(0.868, 1.000, 1.000)	(0.702, 0.934, 1.100)	(0.734, 0.868, 1.000)	(0.680, 0.768, 1.034)	(0.506, 0.680, 0.902)	(1.000, 1.000, 1.000)	(1.000, 1.100, 1.300)	(0.880, 1.000, 1.134)
C7	(0.760, 0.800, 0.968)	(0.734, 0.968, 1.200)	(1.000, 1.000, 1.100)	(0.868, 1.000, 1.100)	(0.460, 0.602, 0.868)	(0.834, 0.934, 1.000)	(1.000, 1.000, 1.000)	(1.100, 1.200, 1.400)
C8	(0.648, 0.834, 0.934)	(0.700, 0.802, 1.100)	(0.900, 1.068, 1.300)	(1.000, 1.100, 1.300)	(0.494, 0.668, 0.868)	(1.000, 1.134, 1.300)	(0.814, 0.900, 0.934)	(1.000, 1.000, 1.000)

**Table 7:** Fuzzy criteria weights ${\tilde{w}}_{\text{criteria}}=\left({\tilde{w}}_{1},{\tilde{w}}_{2},\ldots ,{\tilde{w}}_{n}\right)$ determined with F-AHP.

Criteria	Fuzzy weights
C1	(0.116, 0.168, 0.242)
C2	(0.094, 0.141, 0.214)
C3	(0.079, 0.112, 0.164)
C4	(0.074, 0.107, 0.152)
C5	(0.093, 0.144, 0.212)
C6	(0.079, 0.109, 0.149)
C7	(0.082, 0.110, 0.152)
C8	(0.079, 0.110, 0.153)

**Table 8:** 5 DMs' evaluation scores of the influenza intervention alternatives with respect to each criterion.

	C1	C2	C3	C4	C5	C6	C7	C8
A1	MG	G	F	G	VP	VG	VP	MG
	G	G	MG	MG	MP	VG	G	G
	VG	VG	F	G	MP	F	G	G
	MG	MP	VG	F	G	MP	VG	G
	F	MG	MG	G	MP	G	G	G

A2	VG	G	VG	VG	VG	VG	VG	G
	G	G	VG	VG	G	VG	VG	G
	MP	F	F	MG	G	MG	VG	G
	G	P	P	G	F	MP	G	G
	G	G	G	MG	VG	F	MG	MG

A3	MP	VG	VG	P	MP	F	VP	F
	F	G	G	F	MP	VP	VP	MP
	VG	VG	VG	G	MP	P	VP	P
	G	VG	MP	F	P	F	F	VP
	VP	P	MP	MP	VP	P	P	VP

**Table 9:** Fuzzy evaluation matrix ($\tilde{D}$) for the alternatives and fuzzy best values (FBV) and fuzzy worst values (FWV).

	C1	C2	C3	C4	C5	C6	C7	C8
A1	(5.800, 7.600, 9.000)	(5.800, 7.600, 8.800)	(5.000, 6.800, 8.400)	(5.800, 7.800, 9.200)	(2.000, 3.600, 5.200)	(5.800, 7.400, 8.400)	(6.000, 7.400, 8.200)	(6.600, 8.600, 9.800)
A2	(6.200, 8.000, 9.000)	(4.800, 6.600, 8.000)	(5.600, 7.000, 8.000)	(7.000, 8.600, 9.600)	(7.000, 8.600, 9.400)	(5.400, 7.000, 8.200)	(7.800, 9.200, 9.800)	(6.600, 8.600, 9.800)
A3	(4.000, 5.400, 6.600)	(6.800, 8.000, 8.600)	(5.400, 7.000, 8.000)	(2.800, 4.600, 6.400)	(0.600, 2.000, 3.800)	(1.200, 2.400, 4.200)	(0.600, 1.200, 2.600)	(0.800, 1.800, 3.400)
FBV	(6.200, 8.000, 9.000)	(6.800, 8.000, 8.800)	(5.600, 7.000, 8.400)	(7.000, 8.600, 9.600)	(7.000, 8.600, 9.400)	(5.800, 7.400, 8.400)	(7.800, 9.200, 9.800)	(6.600, 8.600, 9.800)
FWV	(4.000, 5.400, 6.600)	(4.800, 6.600, 8.000)	(5.000, 6.800, 8.000)	(2.800, 4.600, 6.400)	(0.600, 2.000, 3.800)	(1.200, 2.400, 4.200)	(0.600, 1.200, 2.600)	(0.800, 1.800, 3.400)

**Table 10:** ${\tilde{S}}_{i}$, ${\tilde{R}}_{i}$, ${\tilde{S}}^{*},{\tilde{S}}^{-},{\tilde{R}}^{*},\text{ and }{\tilde{R}}^{-}$ values.

	${\tilde{S}}_{i}$	${\tilde{R}}_{i}$	${\tilde{Q}}_{i}$
A1	(−1.744, 0.333, 4.200)	(0.019, 0.112, 1.691)	(−1.133, 0.150, 1.025)
A2	(−1.841, 0.150, 3.379)	(−0.032, 0.141, 1.691)	(−1.050, 0.256, 1.050)
A3	(−1.576, 0.747, 4.404)	(0.047, 0.168, 1.724)	(−1.164, 1.000, 1.000)

${\tilde{S}}^{*}$ =	(−.841, 0.150, 3.379)	${\tilde{R}}^{*}$ = (−0.032, 0.112, 1.691)	
${\tilde{S}}^{-}$ =	(−1.576, 0.747, 4.404)	${\tilde{R}}^{-}$ = (0.047, 0.168, 1.724)	

**Table 11:** Fuzzy AHP-VIKOR results for influenza intervention strategies.

	*S*_*i*_	Rank	*R*_*i*_	Rank	*Q*_*i*_	Rank
A1	0.632	2	0.360	1	0.085	1
A2	0.356	1	0.370	2	0.171	2
A3	0.969	3	0.407	3	0.639	3

## Data Availability

The data used to support the findings of this study are included within the article.

## References

[1] Cauchemez S., Van Kerkhove M. D., Archer B. N. (2014). School closures during the 2009 influenza pandemic: national and local experiences. *BMC Infectious Diseases*.

[2] Kelso J., Halder N., Milne G. (2013). Vaccination strategies for future influenza pandemics: a severity-based cost effectiveness analysis (provisional abstract). *BMC Infectious Diseases*.

[3] European Center for Disease Prevention and Control (2010). The 2009 A(H1N1) pandemic in Europe, reproduction.

[4] Mereckiene J., Cotter S., Weber J. T. (2012). Influenza A(H1N1)pdm09 vaccination policies and coverage in Europe. *Eurosurveillance*.

[5] Samanlioglu F., Bilge A. H. (2016). An overview of the 2009 A(H1N1) pandemic in Europe: efficiency of the vaccination and healthcare strategies. *Journal of Healthcare Engineering*.

[6] Freiesleben de Blasio B., Iversen B. G., Tomba G. (2012). Effect of vaccines and antivirals during the major 2009 A(H1N1) pandemic wave in Norway—and the influence of vaccination timing. *PLoS One*.

[7] Waalen K., Kilander A., Dudman S. G., Krogh G. H., Aune T., Hungnes O. (2010). High prevalence of antibodies to the 2009 pandemic influenza A(H1N1) virus in the Norwegian population following a major epidemic and a large vaccination campaign in autumn 2009. *Eurosurveillance*.

[8] Merler S., Ajelli M., Pugliese A., Ferguson N. M. (2011). Determinants of the spatiotemporal dynamics of the 2009 H1N1 pandemic in Europe: implications for real-time modelling. *PLoS Computational Biology*.

[9] Wilking H., Buda S., von der Lippe E. (2010). Mortality of 2009 pandemic influenza A (H1N1) in Germany. *Eurosurveillance*.

[10] Shin T., Kim C.-B., Ahn Y.-H. (2009). The comparative evaluation of expanded national immunization policies in Korea using an analytic hierarchy process. *Vaccine*.

[11] Mourits M. C. M., van Asseldonk M. A. P. M., Huirne R. B. M. (2010). Multi criteria decision making to evaluate control strategies of contagious animal diseases. *Preventive Veterinary Medicine*.

[12] Aenishaenslin C., Hongoh V., Cissé H. D. (2013). Multi-criteria decision analysis as an innovative approach to managing zoonoses: results from a study on Lyme disease in Canada. *BMC Public Health*.

[13] Pooripussarakul S., Riewpaiboon A., Bishai D., Muangchana C., Tantivess S. (2016). What criteria do decision makers in Thailand use to set priorities for vaccine introduction?. *BMC Public Health*.

[14] Zadeh L. A. (1994). Fuzzy logic, neural networks, and soft computing. *Communications of the ACM*.

[15] Saaty T. L. (1980). *The Analytic Hierarchy Process*.

[16] Weber S. F. (1993). A modified analytic hierarchy process for automated manufacturing decisions. *Interfaces*.

[17] Yurdakul M. (2004). AHP as a strategic decision-making tool to justify machine tool selection. *Journal of Materials Processing Technology*.

[18] Ayağ Z. (2007). A hybrid approach to machine-tool selection through AHP and simulation. *International Journal of Production Research*.

[19] Cho K.-T., Kim S.-M. (2003). Selecting medical devices and materials for development in Korea: the analytic hierarchy process approach. *The International Journal of Health Planning and Management*.

[20] Opricovic S. (1998). Multicriteria optimization of civil engineering systems.

[21] Tzeng G.-H., Teng M.-H., Chen J.-J., Opricovic S. (2002). Multicriteria selection for a restaurant location in Taipei. *International Journal of Hospitality Management*.

[22] Opricovic S., Tzeng G.-H. (2004). Compromise solution by MCDM methods: a comparative analysis of VIKOR and TOPSIS. *European Journal of Operational Research*.

[23] Tzeng G.-H., Lin C.-W., Opricovic S. (2005). Multi-criteria analysis of alternative-fuel buses for public transportation. *Energy Policy*.

[24] Opricovic S., Tzeng G.-H. (2007). Extended VIKOR method in comparison with outranking methods. *European Journal of Operational Research*.

[25] Opricovic S. (2011). Fuzzy VIKOR with an application to water resources planning. *Expert Systems with Applications*.

[26] Wan S.-P., Wang Q.-Y., Dong J.-Y. (2013). The extended VIKOR method for multi-attribute group decision making with triangular intuitionistic fuzzy numbers. *Knowledge-Based Systems*.

[27] Ju Y., Wang A. (2013). Extension of VIKOR method for multi-criteria group decision making problem with linguistic information. *Applied Mathematical Modelling*.

[28] Liu H.-C., You J.-X., Fan X.-J., Chen Y.-Z. (2014). Site selection in waste management by the VIKOR method using linguistic assessment. *Applied Soft Computing*.

[29] Qin J., Liu X., Pedrycz W. (2015). An extended VIKOR method based on prospect theory for multiple attribute decision making under interval type-2 fuzzy environment. *Knowledge-Based Systems*.

[30] Yazici İ., Kahraman C. (2015). VIKOR method using interval type two fuzzy sets. *Journal of Intelligent & Fuzzy Systems*.

[31] Narayanamoorthy S., Geetha S. (2017). Intuitionistic hesitant fuzzy VIKOR method for multi-criteria group decision making. *International Journal of Pure and Applied Mathematics*.

[32] Ayağ Z. (2005). A fuzzy AHP-based simulation approach to concept evaluation in a NPD environment. *IIE Transactions*.

[33] Ayağ Z., Özdemir R. G. (2006). A fuzzy AHP approach to evaluating machine tool alternatives. *Journal of Intelligent Manufacturing*.

[34] Durán O., Aguilo J. (2008). Computer-aided machine-tool selection based on a fuzzy-AHP approach. *Expert Systems with Applications*.

[35] Srichetta P., Thurachon W. (2012). Applying fuzzy analytic hierarchy process to evaluate and select product of notebook computers. *International Journal of Modeling and Optimization*.

[36] Avikal S., Mishra P. K., Jain R. (2014). A fuzzy AHP and PROMETHEE method-based heuristic for disassembly line balancing problems. *International Journal of Production Research*.

[37] Kabir G., Sumi R. S. (2014). Power substation location selection using fuzzy analytic hierarchy process and PROMETHEE: a case study from Bangladesh. *Energy*.

[38] Choudhary D., Shankar R. (2012). An STEEP-fuzzy AHP-TOPSIS framework for evaluation and selection of thermal power plant location: a case study from India. *Energy*.

[39] Sakthivel G., Ilangkumaran M., Nagarajan G., Shanmugam P. (2013). Selection of best biodiesel blend for IC engines: an integrated approach with FAHP-TOPSIS and FAHP-VIKOR. *International Journal of Oil, Gas and Coal Technology*.

[40] Kim Y., Chung E.-S. (2013). Fuzzy VIKOR approach for assessing the vulnerability of the water supply to climate change and variability in South Korea. *Applied Mathematical Modelling*.

[41] Jahan A., Mustapha F., Ismail M. Y., Sapuan S. M., Bahraminasab M. (2011). A comprehensive VIKOR method for material selection. *Materials & Design*.

[42] Su Z.-X. (2011). A hybrid fuzzy approach to fuzzy multi-attribute group decision-making. *International Journal of Information Technology & Decision Making*.

[43] Chang T.-H. (2014). Fuzzy VIKOR method: a case study of the hospital service evaluation in Taiwan. *Information Sciences*.

[44] Wu Z., Ahmad J., Xu J. (2016). A group decision making framework based on fuzzy VIKOR approach for machine tool selection with linguistic information. *Applied Soft Computing*.

[45] Musani S., Jemain A. A. (2015). Ranking schools’ academic performance using a fuzzy VIKOR. *Journal of Physics: Conference Series*.

[46] Awasthi A., Kannan G. (2016). Green supplier development program selection using NGT and VIKOR under fuzzy environment. *Computers & Industrial Engineering*.

[47] Yazdani M., Graeml F. R. (2014). VIKOR and its applications. *International Journal of Strategic Decision Sciences*.

[48] Gul M., Celik E., Aydin N., Taskin Gumus A., Guneri A. F. (2016). A state of the art literature review of VIKOR and its fuzzy extensions on applications. *Applied Soft Computing*.

[49] Shahrasbi A., Shamizanjani M., Alavidoost M. H., Akhgar B. (2017). An aggregated fuzzy model for the selection of a managed security service provider. *International Journal of Information Technology & Decision Making*.

[50] Ali Y., Razi M., De Felice F., Sabir M., Petrillo A. (2019). A VIKOR based approach for assessing the social, environmental and economic effects of “smog” on human health. *Science of the Total Environment*.

[51] Ali Y., Asees Awan M., Bilal M., Khan J., Petrillo A., Ali Khan A. (2019). Risk assessment of China-Pakistan fiber optic project (CPFOP) in the light of multi-criteria decision making (MCDM). *Advanced Engineering Informatics*.

[52] Kaya T., Kahraman C. (2010). Multicriteria renewable energy planning using an integrated fuzzy VIKOR & AHP methodology: the case of Istanbul. *Energy*.

[53] Ilangkumaran M., Sasirekha V., Anojkumar L., Raja M. B. (2012). Machine tool selection using AHP and VIKOR methodologies under fuzzy environment. *International Journal of Modelling in Operations Management*.

[54] Dincer H., Hacioglu U. (2013). Performance evaluation with fuzzy VIKOR and AHP method based on customer satisfaction in Turkish banking sector. *Kybernetes*.

[55] Ghadikolaei A. S., Esbouei S. K., Antucheviciene J. (2014). Applying fuzzy MCDM for financial performance evaluation of Iranian companies. *Technological and Economic Development of Economy*.

[56] Rezaie K., Ramiyani S. S., Nazari-Shirkouhi S., Badizadeh A. (2014). Evaluating performance of Iranian cement firms using an integrated fuzzy AHP-VIKOR method. *Applied Mathematical Modelling*.

[57] Anojkumar L., Ilangkumaran M., Sasirekha V. (2014). Comparative analysis of MCDM methods for pipe material selection in sugar industry. *Expert Systems with Applications*.

[58] Aydin S., Kahraman C. (2014). Vehicle selection for public transportation using an integrated multi criteria decision making approach: a case of Ankara. *Journal of Intelligent & Fuzzy Systems*.

[59] Bhosale V. A., Kant R. (2014). Selection of best knowledge flow practicing organisation using hybrid fuzzy AHP-VIKOR method. *International Journal of Decision Sciences, Risk and Management*.

[60] Arunachalam A. P. S., Idapalapati S., Subbiah S. (2015). Multi-criteria decision making techniques for compliant polishing tool selection. *The International Journal of Advanced Manufacturing Technology*.

[61] Klir G. J., Yuan B. (1995). *Fuzzy Sets and Fuzzy Logic: Theory and Applications*.

[62] Lootsma F. A. (1997). *Fuzzy Logic for Planning and Decision Making, Applied Optimization*.

[63] Yong D. (2005). Plant location selection based on fuzzy TOPSIS. *The International Journal of Advanced Manufacturing Technology*.

[64] Asees Awan M., Ali Y. (2019). Sustainable modeling in reverse logistics strategies using fuzzy MCDM: case of China Pakistan Economic Corridor. *Management of Environmental Quality: An International Journal*.

[65] Chen L. Y., Wang T.-C. (2009). Optimizing partners’ choice in IS/IT outsourcing projects: the strategic decision of fuzzy VIKOR. *International Journal of Production Economics*.

